# Data for high-throughput estimation of specific activities of enzyme/mutants in cell lysates through immunoturbidimetric assay of proteins

**DOI:** 10.1016/j.dib.2017.07.019

**Published:** 2017-07-15

**Authors:** Yiran Feng, Xiaolan Yang, Huimin Chong, Deqiang Wang, Xiaolei Hu, Chang-Guo Zhan, Fei Liao

**Affiliations:** a Unit for Analytical Probes and Protein Biotechnology, Key Laboratory of Medical Laboratory Diagnostics of the Education Ministry, College of Laboratory Medicine, Chongqing Medical University, Chongqing 400016, China; b Molecular Modeling and Biopharmaceutical Center and Department of Pharmaceutical Sciences, College of Pharmacy, University of Kentucky, 789 South Limestone Street, Lexington, KY 40536, USA

**Keywords:** Immunoturbidimetric assay, Specific activity, HTP assay, Mutant, Screen

## Abstract

Data in this article are associated with the research article “Highthroughput estimation of specific activities of enzyme/mutants in cell lysates through immunoturbidimetric assay of proteins” (Yang et al., 2017) [1]. This article provided data on how to develop an immunoturbidimetric assay (ITA) of enzyme/mutants as proteins in cell lysates in high-throughput (HTP) mode together with HTP assay of their activities to derive their specific activities in cell lysates for comparison, with *Pseudomonas aeruginosa* arylsulfatase (PAAS) and *Bacillus fastidious* uricase (BFU) plus their mutants as models. Data were made publicly available for further analyses.

## Specifications Table

**Table:** 

Subject area	Chemistry, Biology
More specific subject area	Biomolecule engineering
Type of data	Table, graph, figure
How data was acquired	Biotek ELX 800 and BIOTEK EON microplate readers to record the adsorption for activity assay, and to record the extinction of ITA complex for selective quantification of protein in 96-well plates
Data format	Raw and analyzed
Experimental factors	Interferences found from denaturated proteins in medium, which are eliminated through filtration of cell lysates through 0.22 μm membrane
Experimental features	Specific activities of enzyme/mutants in cell lysates based on ITA of their proteins with one of the purified enzyme/mutants as the reference protein were compared with those of purified enzyme/mutants by directed assay
Data source location	Chongqing Medical University, Chongqing 400016, China
Data accessibility	Data are available with this article

## Value of the data

• Supporting the validity of ITA of a group of enzyme/mutants as proteins in cell lysates to derive their specific activities for comparison.
• Supporting much higher reliability to recognize a positive mutant of 50% higher activity by the comparison of specific activities based on ITA of enzyme/mutants as proteins in cell lysates than the comparison of other activity indices.
• Supporting the incomparable advantage of cost and labor for the elucidation of sequence-activity relationship of an enzyme.

## 1 Data

**Fig. 1:**
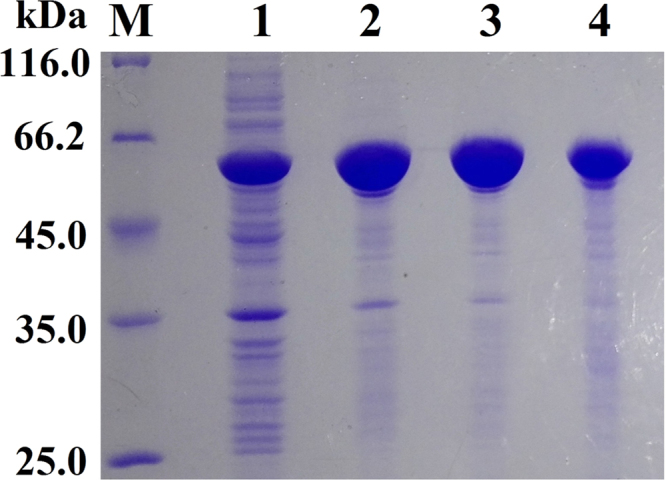
SDS-PAGE analyses of preparations of PAAS. M: molecular weight markers; 1: PAAS after affinity chromatography once; 2: PAAS after affinity chromatography twice; 3: PAAS after affinity chromatography trice; 4: PAAS after affinity chromatography four times. Each lane was loaded the same 15 μg of total proteins by the Bradford assay.

**Fig. 2:**
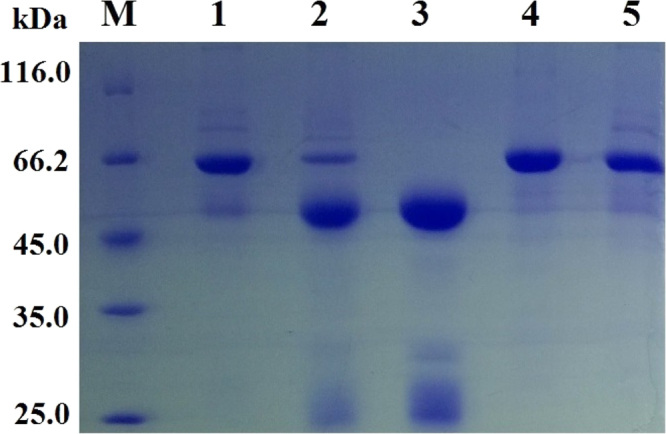
Purification of polyclonal antibodies analyzed by SDS-PAGE. 1. proteins in antisera; 2. proteins in the precipitate by 33% ammonia sulfate at 4 °C, yield 20%; 3. the dissolved precipitate after DEAE-cellulose chromatography at pH 6.5, yield 25%; 4. BSA; 5. Proteins in the supernatant of 33% ammonia sulfate (application data in Table 12).

**Fig. 3:**
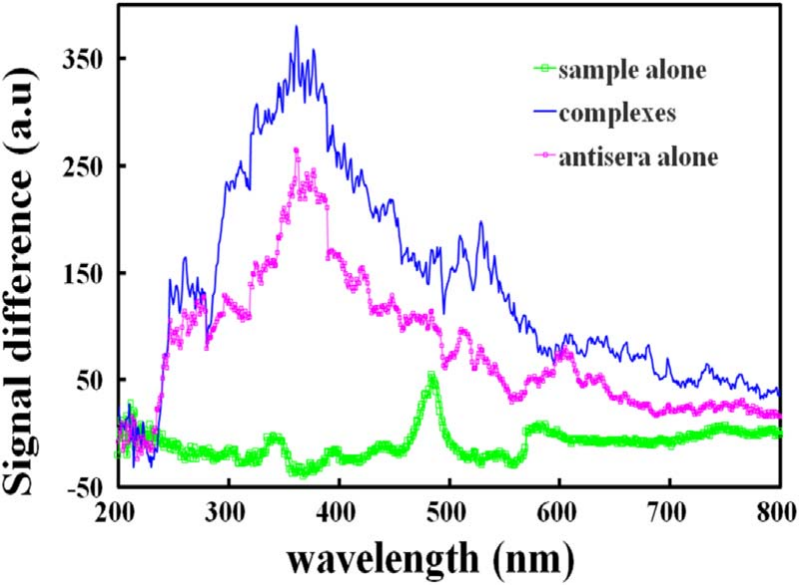
Wavelength effects on scattering signals of reaction mixture containing a sample of 1.0 μg PAAS and antisera of 0.25 mg.

**Fig. 4:**
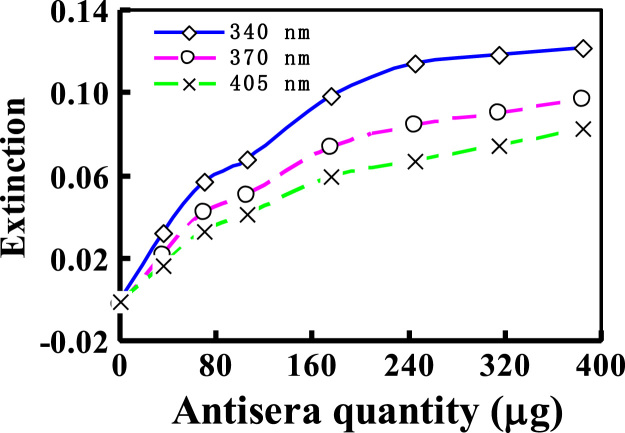
Effects of the combination wavelengths on ITA signals of reaction mixture containing 1.0 μg PAAS plus varying quantities of its antisera.

**Fig. 5:**
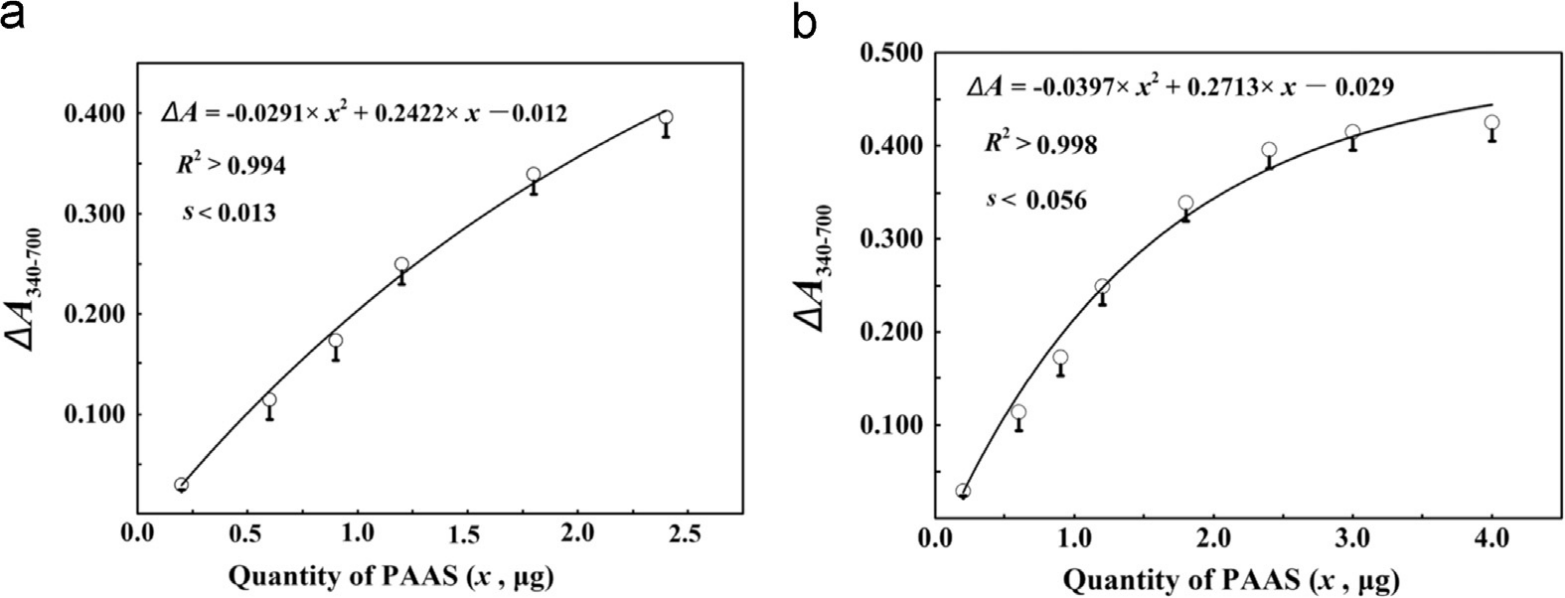
Fitting of a quadratic function to the response of △ΔA_340-700_ to PAAS quantities in mixtures (the same data in Table 13, standard error of estimate was about 0.013 for PAAS from 0.2 to 2.4 μg (a), but was as large as about 0.056 for PAAS from 0.2 to 4.0 μg (b)).

**Fig. 6:**
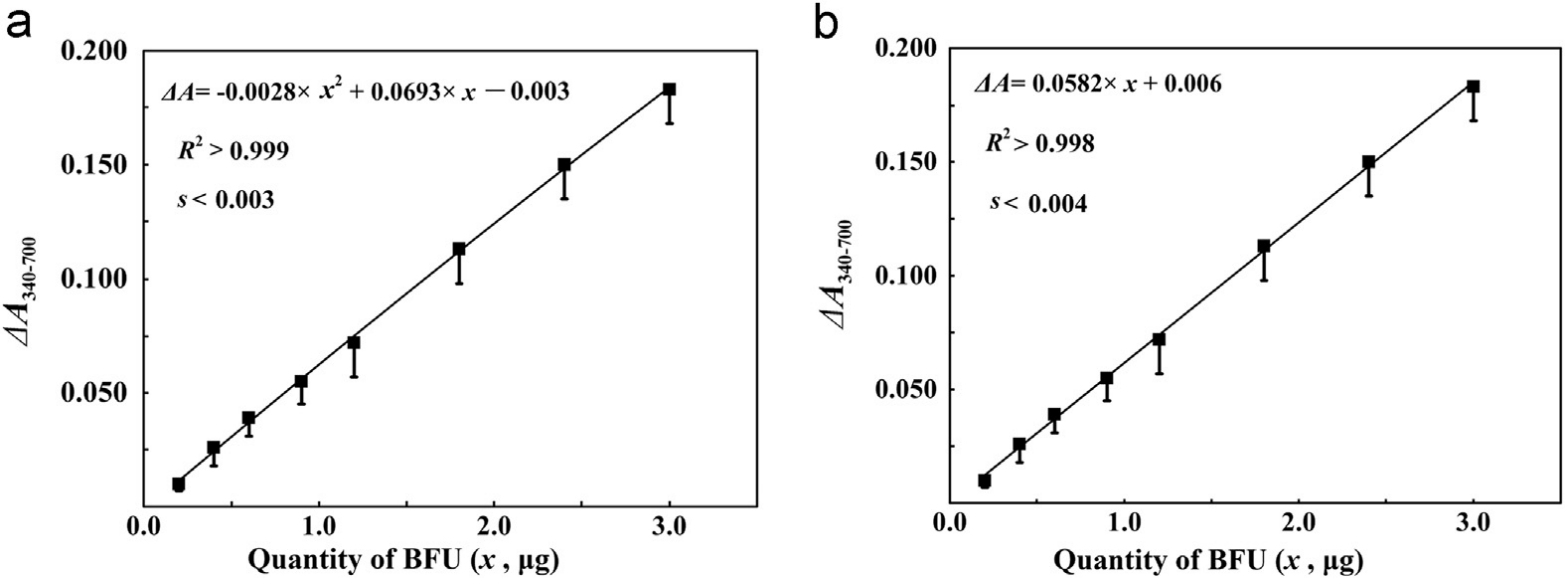
Fitting of two models to the response of *A*_340-700_ to BFU quantities in mixtures (the same data in Table 14, standard error of estimate was about 0.003 for fitting with a quadratic function (a), but was about 0.004 for fitting with a linear function (b), for BFU from 0.20 to 3.0 μg).

**Fig. 7:**
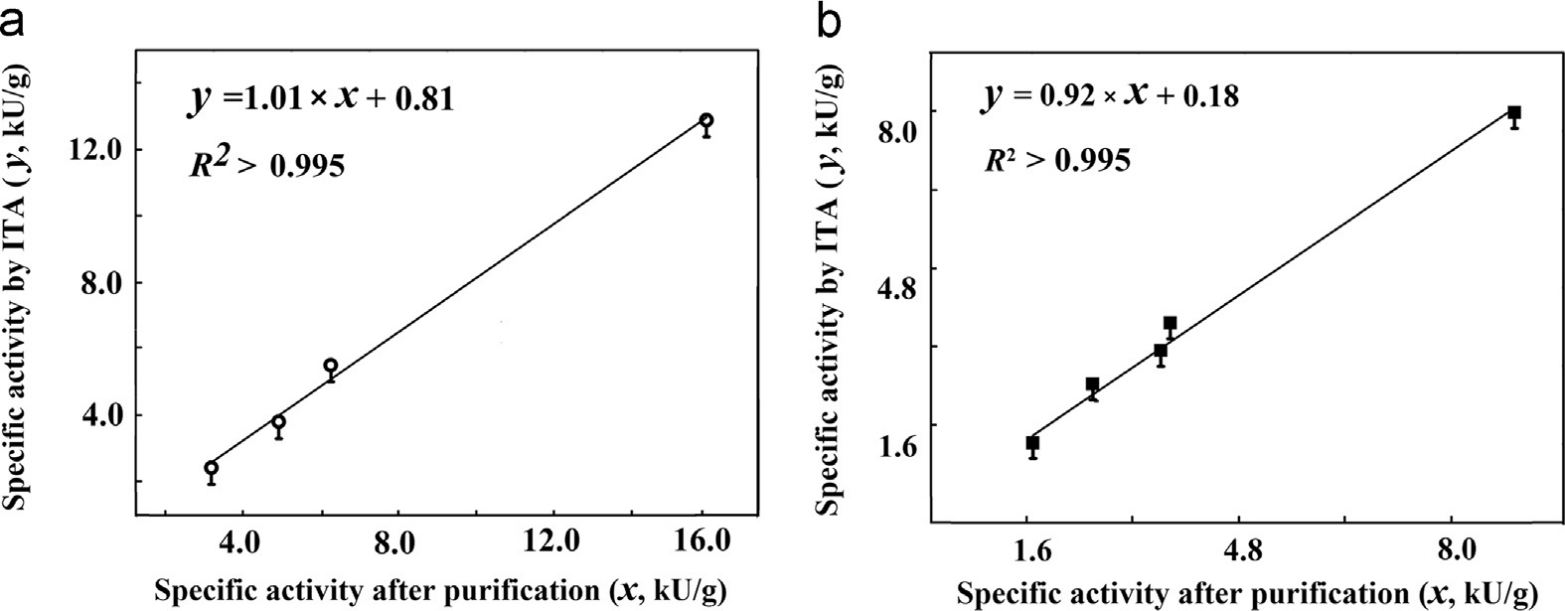
Association of relative specific activities based on ITA with those after purification a, PAAS/mutants; left to right were M72D, G138S, M72Q, and PAAS b, BFU/mutants; left to right were F301L-6H, L171I, A1R, BFU-6H and BFU.

**Fig. 8:**
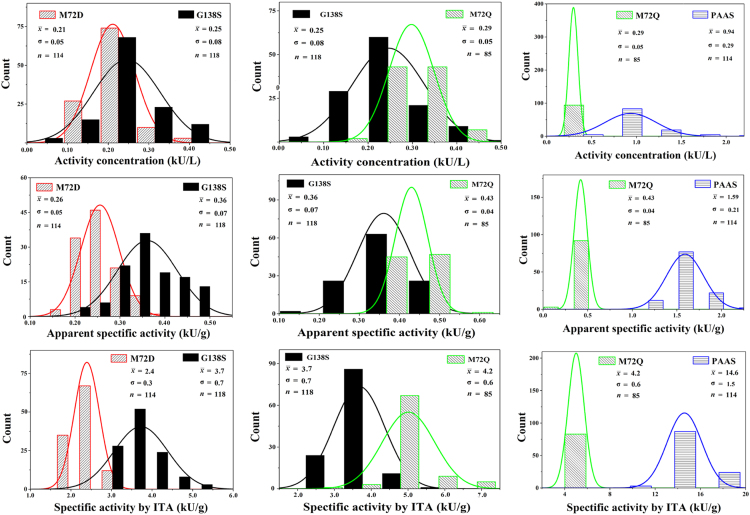
Distributions of specific activities based on ITA of proteins and activity concentrations in each pair of PAAS/mutants.

**Fig. 9:**
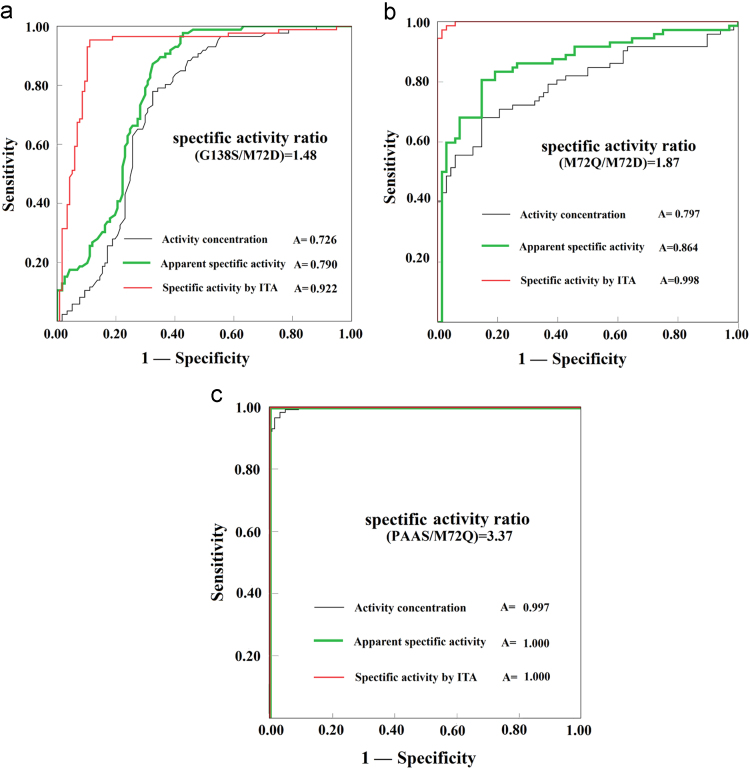
Comparison of the recognition of the positive one in a pair through Receiver-Operating-Characteristic analyses of specific activities based on ITA and other activity indices.

The data in this article provides information on how to develop an experimental system to determine specific activities of enzyme/mutants in cell lysates in HTP mode based on ITA of enzyme/mutants as protein and HTP assay of activities (Fig. 1, Fig. 2, Fig. 3, Fig. 4, Fig. 5, Fig. 6, Fig. 7 and Table 1, Table 2, Table 3, Table 4, Table 5, Table 6, Table 7, Table 8, Table 9, Table 10, Table 11, Table 12, Table 13, Table 14, Table 15, Table 16, Table 17, Table 18, Table 19, Table 20, Table 21, Table 22, Table 23, Table 24, Table 25). Data were provided for the validity of the proposed strategy, the performance to recognize the positive mutant in each random pair of PAAS/mutants during HTP screening, and the efficacy to elucidate sequence-activity relationships of both BFU and PAAS in HTP mode (Fig. 8, Fig. 9 and Table 26).

**Table 1:** Changes of apparent specific activities of PAAS during purification.

Affinity purification of PAAS	(Apparent) specific activity (kU/g, assay in duplicate)	
Cell lysate	2.3±0.2	
The first purification	7.0±0.2	initial rate with data from 10 to 15 min reaction after the mixing of 4NPS with PAAS
The second purification	10.8±0.3	
The third purification	12.6±0.3	
The fourth purification	14.5±0.3	

The four enzymes after affinity purification once were concurrently assumed to have the purity of 48%, to correct the effects of purity on their specific activities. BIOTEK ELX800 reader (data from 10 to 15 min).

**Table 2:** Deviations between systems for M72D activity assay and the correction of its specific activity.

M72D		MAPADA UV 1600 spectrophotometer (data within 1.0 min)		MAPADA UV 1600 spectrophotometer (data from 10 to 15 min)		The ratio of rate within 1.0 min to that from 10 to 15 min, all by MAPADA UV 1600 spectrophotometer	Biotek ELX 800 microplate reader (data from 10 to 15 min)		The ratio of rate within 1.0 min by MAPADA UV to that from 10 to 15 min by BIOTEK ELX 800 microplate reader
		Activity concentration (kU/L)	Apparent specific activity (kU/g)	Activity concentration (kU/L)	Apparent specific activity (kU/g)		Activity concentration (kU/L)	Apparent specific activity (kU/g)	
Repetition		2.333	2.430	1.299	1.353	1.795	0.975	1.060	2.293
		2.222	2.315	1.406	1.465	1.580	1.004	1.092	2.121
		2.284	2.379	1.404	1.463	1.626	0.993	1.079	2.204
		2.364	2.463	1.497	1.559	1.580	0.987	1.073	2.295
		2.281	2.376	1.490	1.552	1.531	1.026	1.115	2.131
		2.288	2.384	1.521	1.584	1.505	1.020	1.109	2.150
		2.286	2.381	1.486	1.548	1.538	1.047	1.138	2.092
		2.462	2.565	1.537	1.601	1.603	1.145	1.244	2.062
		2.262	2.356	1.553	1.618	1.456	1.042	1.132	2.080
		2.460	2.563	1.586	1.652	1.551	0.950	1.032	2.483
Repetition		2.389	2.488	1.585	1.651	1.507			
	Mean	2.330	2.427	1.488	1.550	1.570	1.019	1.107	2.191
	SD	0.080	0.083	0.087	0.091	0.089	0.054	0.058	0.131
	CV	0.034	0.034	0.059	0.059	0.057	0.053	0.053	0.060
Corrected for 48% purity								2.31	
SD for 48% purity								0.12	

The enzyme was purified by Ni^2+^-NTA column and buffer was pre-incubated at room temperature for 30 min prior to use.

**Table 3:** Deviations between systems for M72Q activity assay and the correction of its specific activity.

M72Q		MAPADA UV 1600 spectrophotometer (data within 1.0 min)		MAPADA UV 1600 spectrophotometer (data from 10 to 15 min)		The ratio of rate within 1.0 min to that from 10 to 15 min, all by MAPADA UV 1600 spectrophotometer	Biotek ELX 800 microplate reader (data from 10 to 15 min)		The ratio of rate within 1.0 min by MAPADA UV to that from 10 to 15 min by BIOTEK ELX 800 microplate reader
		Activity concentration (kU/L)	Apparent specific activity (kU/g)	Activity concentration (kU/L)	Apparent specific activity (kU/g)		Activity concentration (kU/L)	Apparent specific activity (kU/g)	
Repetition		3.344	6.079	1.503	2.732	2.225	1.036	1.884	3.227
		2.718	4.942	1.317	2.394	2.064	1.062	1.931	2.558
		3.091	5.620	1.437	2.614	2.150	0.943	1.715	3.276
		3.001	5.456	1.446	2.629	2.075	1.122	2.040	2.675
		3.163	5.750	1.519	2.762	2.082	0.981	1.784	3.223
		3.004	5.462	1.443	2.623	2.083	0.932	1.694	3.224
		3.027	5.504	1.412	2.566	2.144	0.945	1.718	3.203
		2.846	5.175	1.237	2.249	2.301	1.165	2.119	2.442
		3.125	5.682	1.443	2.623	2.166	0.972	1.768	3.213
		3.038	5.524	1.355	2.464	2.242	0.977	1.776	3.110
Repetition		3.001	5.456	1.490	2.709	2.014			
	Mean	3.032	5.514	1.418	2.579	2.141	1.014	1.843	3.015
	SD	0.162	0.295	0.085	0.154	0.088	0.080	0.146	0.322
	CV	0.053	0.053	0.060	0.060	0.041	0.079	0.079	0.107
Corrected for 48% purity								3.84	
SD for 48% purity								0.30	

The enzyme was purified by Ni^2+^-NTA column and buffer was pre-incubated at room temperature for 30 min prior to use.

**Table 4:** Deviations between systems for G138S activity assay and the correction of its specific activity.

G138S		MAPADA UV 1600 spectrophotometer (data within 1.0 min)		MAPADA UV 1600 spectrophotometer (data from 10 to 15 min)		The ratio of rate within 1.0 min to that from 10 to 15 min, all by MAPADA UV 1600 spectrophotometer	Biotek ELX 800 microplate reader (data from 10 to 15 min)		The ratio of rate within 1.0 min by MAPADA UV to that from 10 to 15 min by BIOTEK ELX 800 microplate reader
		Activity concentration (kU/L)	Apparent specific activity (kU/g)	Activity concentration (kU/L)	Apparent specific activity (kU/g)		Activity concentration (kU/L)	Apparent specific activity (kU/g)	
Repetition		7.215	5.228	2.161	1.566	3.339	1.891	1.298	4.027
		6.861	4.971	2.016	1.461	3.403	2.055	1.344	3.700
		6.966	5.048	1.752	1.270	3.976	1.765	1.578	3.199
		7.102	5.146	2.041	1.479	3.480	2.064	1.441	3.571
		8.459	6.130	2.240	1.623	3.777	1.925	1.677	3.655
		7.019	5.086	2.146	1.555	3.270	1.707		
		6.989	5.064	2.276	1.649	3.071	1.832	1.706	2.969
		8.150	5.906	2.353	1.705	3.464	2.296	1.664	3.550
		8.760	6.348	2.417	1.751	3.625	1.900	1.565	4.056
		7.547	5.469	2.461	1.783	3.067	2.422	1.311	4.171
Repetition		8.617	6.244	2.314	1.677	3.725			
	Mean	7.608	5.513	2.198	1.593	3.472	1.986	1.509	3.655
	SD	0.740	0.536	0.206	0.149	0.287	0.228	0.164	0.398
	CV	0.097	0.097	0.094	0.094	0.083	0.115	0.108	0.109
Corrected for 48% purity								3.14	
SD for 48% purity								0.34	

The enzyme was purified by Ni^2+^-NTA column and buffer was pre-incubated at room temperature for 30 min prior to use.

**Table 5:** Deviations between systems for PAAS activity assay and the correction of its specific activity.

PAAS		MAPADA UV 1600 spectrophotometer (data within 1.0 min)		MAPADA UV 1600 spectrophotometer (data from 10 to 15 min)		The ratio of rate within 1.0 min to that from 10 to 15 min, all by MAPADA UV 1600 spectrophotometer	Biotek ELX 800 microplate reader (data from 10 to 15 min)		The ratio of rate within 1.0 min by MAPADA UV to that from 10 to 15 min by BIOTEK ELX 800 microplate reader
		Activity concentration (kU/L)	Apparent specific activity (kU/g)	Activity concentration (kU/L)	Apparent specific activity (kU/g)		Activity concentration (kU/L)	Apparent specific activity (kU/g)	
Repetition		30.398	14.475	10.194	4.854	2.982	8.197	6.675	2.169
		29.252	13.929	10.176	4.846	2.875	8.186	6.860	2.030
		26.568	12.651	9.994	4.759	2.658	8.197	6.562	1.928
		26.508	12.623	9.710	4.624	2.730	9.165	7.632	1.654
		27.292	12.996	9.763	4.649	2.795	8.441	6.715	1.935
		28.287	13.470	8.898	4.237	3.179	7.849	6.350	2.121
		27.955	13.312	9.801	4.667	2.852	8.499	7.202	1.848
		25.150	11.976	9.207	4.384	2.732	9.704	7.997	1.498
		25.150	11.976	9.207	4.384	2.732	7.252	6.683	1.792
		25.000	11.905	9.300	4.429	2.688	8.359	6.990	1.703
Repetition		26.176	12.465	8.553	4.073	3.060			
	Mean	27.067	12.889	9.528	4.537	2.844	8.385	6.967	1.868
	SD	1.765	0.841	0.534	0.254	0.167	0.671	0.510	0.212
	CV	0.065	0.065	0.056	0.056	0.059	0.080	0.073	0.114
Corrected for 48% purity								14.51	
SD for 48% purity								1.06	

The enzyme was purified by Ni^2+^-NTA column and buffer was pre-incubated at room temperature for 30 min prior to use.

**Table 6:** Correction of the inhibition of Ni^2+^ on PAAS/mutants.

Final NiSO_4_ at 25 μM Specific activity after correction of the effect of purity only	Specific activity after correction of inhibition by Ni^2+^			Inhibition percentage % (*n*=4)
PAAS	14.5±1.0	14.5±1.0	<5	Insignificant
M72Q	3.8±0.3	4.3±0.4	14±3	significant
G138S	3.1±0.3	3.4±0.4	10±3	significant
M72D	2.3±0.1	2.3±0.1	<4	Insignificant

Specific activity by BIOTEK ELX800 microplate reader with just 0.20 mL reaction mixture at room temperature; initial rate was determined with data from 10 to 15 min after agitation for 5 min.

**Table 7:** Correction of the specific activities of BFU/mutants.

After correction of purity			Before correction of purity		
BFU		9.0	5.2	58%	*n*=10
BFU-6H		3.5	2.0	58%	*n*=8
A1R		3.3	1.9	58%	*n*=5
F301L-6H		1.6	0.9	58%	*n*=3
L171I		2.6	1.5	58%	*n*=4

Specific activity of BFU was assumed to 9.0 kU/g to approximate its purity after DEAE-cellulose chromatography twice and such purity was assigned to that of other mutants for the correction of their specific activities. Activities were determined with BioTek Eon by absorbance of uric acid at 293 nm.

**Table 8:** Optimization of antisera for ITA to 1.0 μg purified PAAS.

PAAS at final 1.0 μg	Final quantity of antisera (μg)							
	0	35	70	105	175	245	315	385
deltaA (340 nm vs 700 nm)	−0.000	0.032	0.057	0.068	0.099	0.114	0.119	0.122
deltaA (370 nm vs 700 nm)	−0.002	0.022	0.042	0.051	0.073	0.085	0.090	0.097
deltaA (405 nm vs 700 nm)	−0.001	0.017	0.033	0.041	0.059	0.067	0.075	0.083

Signals were recorded with BIOTEK EON microplate reader with 96-well plates.

**Table 9:** Optimization of antisera for ITA to 1.0 μg purified BFU.

BFU at final 1.0 μg	Final quantity of antisera (μg)							
	0	35	70	105	175	245	315	385
deltaA (340 nm vs 700 nm)	−0.000	0.022	0.045	0.067	0.112	0.156	0.169	0.182

Signals were recorded on BIOTEK EON microplate reader with 96-well plates

**Table 10:** ITA with 0.75 mg antisera to determine PAAS in artificial samples of different abundance.

*n*	PAAS	340 nm	700 nm	*A*_340-700_	*ΔA*_340-700_	Mean	SD	CV (%)
3% abundance calculated with known specific activity of 14.5 kU/g with data from 10 to 15 min								
Triplicate	0.6 μg	0.857	0.184	0.674	0.201	0.209*	0.007	3.3
	0.6 μg	0.871	0.185	0.686	0.213			
	0.6 μg	0.870	0.184	0.686	0.213			
Triplicate	1.1 μg	0.941	0.199	0.742	0.261	0.269*	0.007	2.6
	1.1 μg	0.946	0.200	0.746	0.273			
	1.1 μg	0.947	0.201	0.746	0.273			
Triplicate	2.2 μg	1.027	0.219	0.808	0.335	0.350*	0.018	5.1
	2.2 μg	1.078	0.235	0.843	0.370			
	2.2 μg	1.045	0.227	0.818	0.345			

50% abundance calculated with known specific activity of 14.5 kU/g within data from 10 to 15 min								
Triplicate	0.6 μg	0.846	0.173	0.673	0.200	0.196	0.013	6.8
	0.6 μg	0.825	0.169	0.656	0.183			
	0.6 μg	0.840	0.172	0.668	0.195			
Triplicate	1.1 μg	0.907	0.187	0.720	0.247	0.251	0.008	3.2
	1.1 μg	0.909	0.190	0.719	0.246			
	1.1 μg	0.927	0.194	0.733	0.260			
Triplicate	2.2 μg	1.060	0.225	0.835	0.362	0.350	0.011	3.1
	2.2 μg	1.040	0.225	0.815	0.342			
	2.2 μg	1.043	0.225	0.818	0.345			

There were about additional 14 μg host proteins with the sample of 3% abundance of PAAS in comparison of that with the sample of 50% abundance. The background with lysates of untransformed cells gave *A*_340-700_ of 0.473. And *t*-test indicated insignificant differences for the same quantities of PAAS but different abundance in artificial cell lysates, as indicated as *.

**Table 11:** ITA with 0.75 mg antisera to determine BFU in artificial samples of different abundance.

*n*	BFU	340 nm	700 nm	*A*_340-700_	*ΔA*_340-700_	Mean	SD	CV (%)
3% abundance calculated with known specific activity of 14.5 kU/g with data from 10 to 15 min								
Triplicate	1.1 μg	0.484	0.104	0.380	0.070	0.073*	0.007	9.6
	1.1 μg	0.486	0.095	0.391	0.081			
	1.1 μg	0.482	0.104	0.378	0.068			

50% abundance calculated with known specific activity of 14.5 kU/g within data from 10 to 15 min								
Triplicate	1.1 μg	0.479	0.101	0.378	0.068	0.070	0.006	8.7
	1.1 μg	0.477	0.092	0.385	0.075			
	1.1 μg	0.480	0.102	0.378	0.068			

There were additional 14 μg host proteins with the sample of 3% abundance of PAAS in comparison of that with the sample of 50% abundance of PAAS. The background with lysates of untransformed cells gave *A*_340-700_ of 0.310. And *t*-test indicated insignificant differences for the same quantity of PAAS but different abundance in artificial cell lysates, which was indicated as *.

**Table 12:** Effects of purified polyclonal antibodies on ITA.

Total protein (PAAS, μg)		0.3	0.6	1.0	1.5	2.0	3.0	4.5

Δ*A*_340__-__700_	Antisera (0.75 mg)	0.028±0.003	0.089±0.008	0.117±0.010	0.155±0.013	0.185±0.013	0.240±0.015	0.320±0.017
	Ammonia sulfate fraction (0.35 mg)	0.024±0.002	0.053±0.005	0.054±0.005	0.130±0.011	0.160±0.012	0.224±0.015	0.260±0.017
	DEAE cellulose purification (0.15 mg)	0.019±0.002	0.041±0.004	0.068±0.005	0.098±0.012	0.112±0.012	0.168±0.013	0.238±0.013
	DEAE cellulose purification (0.35 mg)	0.049±0.004	0.104±0.011	0.148±0.011	0.214±0.017	0.255±0.010	0.370±0.015	0.536±0.017
							
Total protein (BFU, μg)		0.3	0.5	1.0	1.7	2.1	2.8	4.2

Δ*A*_340__-__700_	Antisera (0.75 mg)	0.018±0.002	0.042±0.004	0.067±0.006	0.101±0.009	0.114±0.010	0.134±0.010	0.182±0.012
	Ammonia sulfate fraction (0.35 mg)	0.021±0.002	0.028±0.003	0.054±0.005	0.087±0.009	0.098±0.009	0.124±0.010	0.150±0.012
	DEAE cellulose purification (0.15 mg)	0.013±0.002	0.022±0.003	0.038±0.004	0.064±0.006	0.072±0.007	0.091±0.008	0.135±0.012
	DEAE cellulose purification (0.35 mg)	0.029±0.003	0.052±0.004	0.088±0.006	0.143±0.011	0.158±0.012	0.200±0.013	0.300±0.014

**Table 13:** Fitting of a quadratic function to response of ΔA340-700 to PAAS quantities in reaction mixtures.

PAAS (μg)	*A*_340-700_		*ΔA* (_340–700_)	CV	A quadratic model (0.4–2.4)			A quadratic model (0.4–4.0)		
	(n=3)				calculate Y	Residual dY		calculate Y	Residual dY	
0.0		0.603	0.000		−0.012	−0.012		−0.029	−0.029	
0.2		0.632	0.029	49.5	0.036	0.007		0.024	0.005	
0.4		0.671	0.071	28.5	0.081	0.010	LOQ	0.073	0.002	LOQ
0.6		0.717	0.114	24.2	0.123	0.009		0.119	0.005	
0.9		0.776	0.173	14.8	0.183	0.010		0.183	0.010	
1.2		0.852	0.249	8.2	0.237	−0.012		0.239	−0.010	
1.8		0.942	0.339	4.2	0.330	−0.009		0.331	−0.008	
2.4		0.999	0.396	5.5	0.402	0.006	UOQ	0.393	−0.003	
3.0		1.018	0.415	9.3	0.453	0.038		0.428	0.013	
4.0		1.028	0.425	9.3	0.492	0.067		0.421	−0.004	UOQ
Buffer alone		0.067			*s*	0.013		*s*	0.056	
Antisera alone		0.552	The fitting with a quadratic function gave *s* < 0.013 and *R*^2^ > 0.994 for UOQ of 2.4 μg.							
Parameters for fitting with a linear model										
Range	Data for 0~1.2 μg					Data for 0~2.4 μg				
	slope	intercept	correl	steyx		slope	correl	intercept	steyx	
	0.208	−0.009	0.998	0.007		0.174	0.982	0.008	0.021

**Table 14:** Fitting of a function to response of Δ*A*_340-700_ to BFU quantities in reaction mixtures.

Quantity	*A*340-700	*ΔA*_(__340-700__)_	a quadratic model				a linear model		
BFU (μg)	(*n*=3)		CV	Calculated *Y*	Residual d*Y*		Calculated *Y*	Residual d*Y*	
0.0	0.315	0.000		−0.003	−0.003		0.007	0.007	
0.2	0.325	0.010	32.5	0.011	0.001		0.018	0.008	
0.4	0.341	0.026	14.5	0.025	−0.001	LOQ	0.030	0.004	LOQ
0.6	0.354	0.039	11.2	0.038	−0.001		0.041	0.002	
0.9	0.370	0.055	9.5	0.058	0.003		0.059	0.004	
1.2	0.387	0.072	6.2	0.077	0.005		0.076	0.004	
1.8	0.428	0.113	5.2	0.113	0.000		0.111	−0.002	
2.4	0.465	0.150	6.5	0.148	−0.002		0.146	−0.002	
3.0	0.498	0.183	5.3	0.180	−0.003	UOQ	0.181	−0.003	UOQ
4.0	0.512	0.197	6.2	0.230	0.038		0.239	0.042	
Buffer alone	0.097								
Antisera alone	0.312			*s*	0.003		*s*	0.004	
A quadratic curve					A linear curve				
Range	0.0–3.0 μg				Range	0.0–3.0 μg			
Slope	intercept	correl	steyx		Slope	correl	intercept	steyx	
0.060	-0.003	0.998	0.003		0.058	0.998	0.006	0.004

**Table 15:** Abundance of M72D determined in HTP mode for comparison (CBB stands for the Bradford assay).

No.	M72D Activity (kU/L)	M72D quantity by CBB for total proteins (g/L)	M72D by ITA (g/L)	M72D Specific activity based on ITA (kU/g)	M72D apparent specific activity by CBB (kU/g)	M72D abundance by activity (its known specific activity used)	M72D abundance by ITA
1	0.187	0.810	0.090	2.084	FALSE	0.101	0.111
2	0.204	0.816	0.104	1.966	0.250	0.108	0.127
3	0.175	0.780	0.085	2.061	0.224	0.097	0.109
4	0.214	0.882	0.106	2.024	0.243	0.105	0.120
5	0.166	0.740	0.069	2.414	0.225	0.098	0.093
6	0.163	0.725	0.066	2.452	0.224	0.097	0.091
7	0.223	0.777	0.103	2.162	0.287	0.125	0.133
8	0.204	0.757	0.101	2.016	0.270	0.117	0.134
9	0.201	0.752	0.100	2.015	0.267	0.116	0.133
10	0.214	0.881	0.107	1.992	0.243	0.106	0.122
11	0.210	0.854	0.093	2.249	0.246	0.107	0.109
14	0.162	0.721	0.073	2.209	0.225	0.098	0.102
15	0.148	0.750	0.063	2.338	0.197	0.086	0.084
16	0.213	0.895	0.101	2.118	0.238	0.104	0.112
17	0.181	0.806	0.080	2.275	0.225	0.098	0.099
18	0.226	0.857	0.118	1.922	0.264	0.115	0.137
19	0.245	0.976	0.123	1.996	0.251	0.109	0.126
20	0.251	0.870	0.112	2.245	0.289	0.125	0.129
21	0.223	0.912	0.112	2.000	0.245	0.107	0.123
22	0.157	0.813	0.066	2.383	0.192	0.084	0.081
23	0.244	0.854	0.122	2.001	0.286	0.124	0.143
24	0.248	0.858	0.120	2.059	0.289	0.126	0.140
25	0.229	0.842	0.107	2.135	0.272	0.118	0.128
26	0.168	0.848	0.074	2.258	0.198	0.086	0.088
27	0.123	0.578	0.070	1.756	0.212	0.092	0.121
28	0.183	0.890	0.073	2.504	0.206	0.089	0.082
29	0.184	0.828	0.071	2.572	0.222	0.096	0.086
30	0.193	0.894	0.078	2.464	0.216	0.094	0.088
31	0.150	0.646	0.066	2.273	0.232	0.101	0.102
32	0.211	0.895	0.084	2.524	0.236	0.103	0.093
33	0.227	0.910	0.104	2.171	0.249	0.108	0.115
34	0.186	0.821	0.078	2.387	0.227	0.099	0.095
35	0.187	0.818	0.073	2.546	0.228	0.099	0.090
36	0.171	0.827	0.066	2.573	0.206	0.090	0.080
37	0.184	0.836	0.072	2.552	0.220	0.096	0.086
38	0.213	0.897	0.098	2.182	0.238	0.103	0.109
39	0.188	0.810	0.073	2.571	0.232	0.101	0.090
40	0.229	0.994	0.092	2.499	0.230	0.100	0.092
41	0.207	0.846	0.084	2.466	0.245	0.106	0.099
42	0.158	0.785	0.065	2.430	0.201	0.087	0.083
43	0.195	0.813	0.074	2.622	0.240	0.104	0.091
44	0.190	0.882	0.075	2.549	0.216	0.094	0.085
45	0.217	0.869	0.084	2.594	0.249	0.108	0.096
46	0.249	0.906	0.110	2.257	0.275	0.119	0.122
47	0.193	0.854	0.081	2.388	0.226	0.098	0.095
48	0.271	1.080	0.123	2.194	0.251	0.109	0.114
49	0.220	0.976	0.084	2.616	0.225	0.098	0.086
50	0.193	0.821	0.087	2.210	0.235	0.102	0.106
51	0.249	0.813	0.107	2.324	0.306	0.133	0.132
52	0.201	0.912	0.081	2.487	0.220	0.096	0.089
53	0.153	0.654	0.061	2.499	0.233	0.101	0.093
54	0.185	0.702	0.090	2.053	0.263	0.114	0.128
55	0.189	0.740	0.090	2.111	0.256	0.111	0.121
56	0.217	0.872	0.088	2.465	0.249	0.108	0.101
57	0.202	0.849	0.096	2.098	0.238	0.103	0.113
58	0.189	0.753	0.075	2.515	0.252	0.109	0.100
59	0.209	0.779	0.073	2.856	0.268	0.116	0.094
60	0.196	0.823	0.093	2.113	0.238	0.103	0.112
61	0.190	0.684	0.075	2.544	0.278	0.121	0.109
62	0.202	0.770	0.100	2.026	0.262	0.114	0.129
63	0.221	0.793	0.081	2.711	0.278	0.121	0.103
64	0.204	0.768	0.078	2.605	0.266	0.116	0.102
65	0.209	0.753	0.075	2.794	0.278	0.121	0.099
66	0.238	0.753	0.099	2.409	0.316	0.138	0.131
67	0.247	0.765	0.098	2.505	0.323	0.140	0.129
68	0.211	0.761	0.084	2.519	0.277	0.120	0.110
69	0.225	0.784	0.092	2.430	0.286	0.125	0.118
70	0.211	0.709	0.084	2.506	0.298	0.130	0.119
71	0.182	0.733	0.095	1.903	0.248	0.108	0.130
72	0.204	0.710	0.068	3.008	0.287	0.125	0.095
73	0.203	0.700	0.081	2.524	0.290	0.126	0.115
74	0.240	0.805	0.110	2.188	0.298	0.130	0.136
75	0.275	0.904	0.106	2.595	0.304	0.132	0.117
76	0.275	0.975	0.142	1.933	0.282	0.123	0.146
77	0.165	0.668	0.084	1.974	0.247	0.107	0.125
78	0.210	0.835	0.078	2.691	0.252	0.109	0.094
79	0.197	0.751	0.073	2.681	0.262	0.114	0.098
80	0.206	0.789	0.096	2.142	0.261	0.114	0.122
81	0.266	0.793	0.098	2.726	0.336	0.146	0.123
82	0.321	0.995	0.123	2.612	0.323	0.140	0.124
83	0.253	0.916	0.092	2.765	0.276	0.120	0.100
84	0.213	0.876	0.084	2.536	0.243	0.106	0.096
85	0.242	0.858	0.085	2.853	0.282	0.123	0.099
86	0.188	0.772	0.074	2.527	0.243	0.106	0.096
87	0.226	0.733	0.075	3.028	0.308	0.134	0.102
88	0.292	0.953	0.123	2.365	0.306	0.133	0.129
89	0.323	1.027	0.124	2.609	0.315	0.137	0.121
90	0.255	0.974	0.107	2.385	0.262	0.114	0.110
Mean	0.210	0.823	0.090	2.362	0.255	0.111	0.109
*σ*	0.036	0.090	0.018	0.272	0.032	0.014	0.017
CV%	0.172	0.110	0.197	0.115	0.127	0.127	0.157

**Table 16:** Abundance of G138S determined in HTP mode for comparison (CBB stands for the Bradford assay).

No.	G138S Activity (kU/L)	G138S quantity by CBB for total proteins (g/L)	G138S quantity by ITA (g/L)	G138S Specific activity based on ITA (kU/g)	G138S Apparent specific activity by CBB (kU/g)	G138S abundance by activity (its known specific activity used)	G138S abundance by ITA
1	0.343	0.929	0.104	3.315	0.370	0.109	0.112
2	0.306	0.678	0.085	3.613	0.452	0.133	0.125
3	0.236	0.509	0.106	2.229	0.463	0.136	0.208
4	0.224	0.545	0.069	3.252	0.411	0.121	0.126
5	0.230	0.494	0.066	3.468	0.466	0.137	0.134
6	0.335	0.757	0.103	3.243	0.443	0.130	0.136
7	0.376	0.951	0.101	3.716	0.396	0.116	0.107
8	0.339	0.755	0.100	3.398	0.449	0.132	0.132
9	0.385	0.872	0.107	3.581	0.441	0.130	0.123
10	0.315	0.709	0.093	3.376	0.444	0.131	0.132
11	0.280	0.607	0.073	3.818	0.461	0.136	0.121
12	0.213	0.555	0.056	3.821	0.384	0.113	0.100
13	0.226	0.563	0.063	3.568	0.401	0.118	0.112
14	0.342	0.851	0.101	3.396	0.401	0.118	0.118
15	0.390	0.886	0.118	3.317	0.440	0.129	0.133
16	0.383	0.937	0.123	3.120	0.409	0.120	0.131
17	0.274	0.591	0.066	4.173	0.464	0.136	0.111
18	0.440	0.964	0.122	3.603	0.457	0.134	0.127
19	0.357	0.977	0.107	3.324	0.366	0.108	0.110
20	0.242	0.723	0.074	3.256	0.335	0.099	0.103
21	0.241	0.731	0.073	3.296	0.330	0.097	0.100
22	0.181	0.591	0.053	3.444	0.306	0.090	0.089
23	0.206	0.604	0.055	3.757	0.340	0.100	0.091
24	0.217	0.660	0.060	3.635	0.329	0.097	0.090
25	0.210	0.680	0.071	2.941	0.309	0.091	0.105
26	0.238	0.632	0.078	3.031	0.376	0.111	0.124
27	0.253	0.680	0.066	3.841	0.373	0.110	0.097
28	0.257	0.830	0.084	3.075	0.310	0.091	0.101
29	0.291	0.834	0.104	2.785	0.349	0.103	0.125
30	0.266	0.793	0.078	3.399	0.335	0.098	0.098
31	0.266	0.764	0.073	3.627	0.348	0.102	0.096
32	0.244	0.841	0.066	3.685	0.290	0.085	0.079
33	0.235	0.713	0.072	3.250	0.329	0.097	0.101
34	0.292	0.823	0.098	2.987	0.354	0.104	0.119
35	0.261	0.797	0.073	3.583	0.328	0.097	0.092
36	0.300	0.805	0.092	3.278	0.373	0.110	0.114
37	0.279	0.821	0.084	3.320	0.339	0.100	0.102
38	0.247	0.800	0.065	3.805	0.308	0.091	0.081
39	0.271	0.876	0.074	3.649	0.310	0.091	0.085
40	0.247	0.760	0.075	3.307	0.325	0.095	0.098
41	0.305	0.808	0.084	3.654	0.378	0.111	0.103
42	0.259	0.760	0.081	3.200	0.340	0.100	0.106
43	0.435	0.954	0.123	3.521	0.455	0.134	0.129
44	0.268	0.820	0.084	3.190	0.327	0.096	0.102
45	0.272	0.785	0.087	3.107	0.346	0.102	0.111
46	0.252	0.780	0.081	3.118	0.323	0.095	0.104
47	0.246	0.591	0.090	2.736	0.416	0.122	0.152
48	0.214	0.543	0.064	3.358	0.393	0.116	0.117
49	0.202	0.586	0.078	2.605	0.345	0.101	0.132
50	0.186	0.594	0.053	3.492	0.312	0.092	0.089
51	0.212	0.603	0.050	4.240	0.352	0.103	0.083
52	0.200	0.586	0.051	3.907	0.341	0.100	0.087
53	0.204	0.591	0.053	3.864	0.346	0.102	0.089
54	0.189	0.589	0.056	3.382	0.320	0.094	0.095
55	0.181	0.587	0.066	2.732	0.308	0.091	0.113
56	0.153	0.589	0.047	3.269	0.260	0.077	0.080
57	0.212	0.591	0.053	3.982	0.359	0.106	0.090
58	0.200	0.758	0.051	3.899	0.264	0.078	0.068
59	0.211	0.647	0.070	3.025	0.326	0.096	0.108
60	0.213	0.614	0.057	3.703	0.347	0.102	0.094
61	0.179	0.594	0.053	3.349	0.300	0.088	0.090
62	0.165	0.742	0.064	2.554	0.222	0.065	0.087
63	0.201	0.609	0.056	3.610	0.331	0.097	0.092
64	0.212	0.739	0.063	3.355	0.287	0.084	0.086
65	0.179	0.818	0.054	3.313	0.219	0.064	0.066
66	0.184	0.640	0.051	3.640	0.288	0.085	0.079
67	0.220	0.703	0.050	4.422	0.312	0.092	0.071
68	0.232	0.714	0.068	3.437	0.325	0.096	0.095
69	0.289	0.700	0.073	3.973	0.414	0.122	0.104
70	0.246	0.862	0.062	3.974	0.285	0.084	0.072
71	0.231	0.647	0.072	3.216	0.357	0.105	0.111
72	0.229	0.710	0.065	3.525	0.322	0.095	0.091
73	0.254	0.789	0.054	4.671	0.322	0.095	0.069
74	0.199	0.974	0.059	3.360	0.204	0.060	0.061
75	0.315	0.782	0.066	4.798	0.403	0.118	0.084
76	0.261	0.925	0.072	3.613	0.282	0.083	0.078
77	0.302	0.786	0.060	5.012	0.384	0.113	0.077
78	0.374	0.937	0.107	3.483	0.399	0.117	0.115
79	0.362	1.046	0.074	4.861	0.346	0.102	0.071
80	0.347	0.754	0.087	3.976	0.461	0.135	0.116
Mean	0.260	0.734	0.075	3.505	0.105	0.105	0.103
*σ*	0.064	0.130	0.020	0.488	0.018	0.018	0.023
CV	0.246	0.177	0.261	0.139	0.170	0.170	0.221

**Table 17:** Abundance of M72Q determined in HTP mode for comparison (CBB stands for the Bradford assay).

No.	M72Q Activity (kU/L)	M72Q by CBB for total proteins (g/L)	M72Q protein by ITA (g/L)	M72Q specific activity based on ITA (kU/g)	M72Q apparent specific activity by CBB (kU/g)	M72Q abundance by activity (its known specific activity used)	M72Q abundance by ITA
1	0.298	0.688	0.073	4.075	0.433	0.101	0.106
2	0.314	0.661	0.111	2.827	0.475	0.110	0.168
3	0.445	0.834	0.106	4.190	0.533	0.124	0.127
4	0.414	0.883	0.105	3.947	0.468	0.109	0.119
5	0.398	0.778	0.098	4.064	0.511	0.119	0.126
6	0.306	0.693	0.114	2.696	0.441	0.103	0.164
7	0.458	0.889	0.118	3.889	0.516	0.120	0.133
8	0.300	0.779	0.076	3.925	0.385	0.090	0.098
9	0.241	0.567	0.104	2.321	0.425	0.099	0.183
10	0.283	0.651	0.075	3.761	0.435	0.101	0.116
11	0.294	0.701	0.063	4.655	0.419	0.098	0.090
12	0.283	0.683	0.070	4.059	0.415	0.096	0.102
13	0.288	0.708	0.078	3.709	0.406	0.094	0.110
14	0.363	0.731	0.085	4.273	0.496	0.115	0.116
15	0.180	0.417	0.046	3.900	0.431	0.100	0.111
16	0.195	0.441	0.054	3.603	0.442	0.103	0.123
17	0.391	0.829	0.088	4.443	0.471	0.110	0.106
18	0.252	0.676	0.064	3.943	0.373	0.087	0.095
19	0.317	0.708	0.079	3.992	0.447	0.104	0.112
20	0.262	0.680	0.064	4.082	0.384	0.089	0.094
21	0.255	0.689	0.058	4.408	0.369	0.086	0.084
22	0.285	0.681	0.052	5.489	0.418	0.097	0.076
23	0.280	0.667	0.066	4.257	0.420	0.098	0.099
24	0.304	0.718	0.080	3.782	0.424	0.099	0.112
25	0.250	0.598	0.061	4.117	0.419	0.097	0.102
26	0.271	0.673	0.076	3.553	0.402	0.094	0.113
27	0.251	0.609	0.062	4.075	0.412	0.096	0.101
28	0.338	0.746	0.083	4.075	0.453	0.105	0.111
29	0.346	0.760	0.081	4.262	0.456	0.106	0.107
30	0.271	0.610	0.062	4.335	0.444	0.103	0.102
31	0.382	0.807	0.100	3.837	0.474	0.110	0.123
32	0.297	0.705	0.081	3.676	0.421	0.098	0.114
33	0.381	0.845	0.112	3.391	0.451	0.105	0.133
34	0.270	0.701	0.065	4.131	0.385	0.089	0.093
35	0.245	0.734	0.060	4.069	0.334	0.078	0.082
36	0.339	0.743	0.087	3.887	0.456	0.106	0.117
37	0.281	0.795	0.066	4.247	0.354	0.082	0.083
38	0.343	0.673	0.060	5.681	0.510	0.119	0.090
39	0.311	0.743	0.057	5.480	0.419	0.097	0.076
40	0.337	0.827	0.084	4.016	0.408	0.095	0.102
41	0.325	0.865	0.081	3.989	0.375	0.087	0.094
42	0.324	0.779	0.060	5.366	0.416	0.097	0.077
43	0.221	0.533	0.057	3.865	0.414	0.096	0.107
44	0.293	0.749	0.068	4.312	0.391	0.091	0.091
45	0.294	0.715	0.077	3.816	0.411	0.096	0.108
46	0.331	0.645	0.080	4.154	0.512	0.119	0.123
47	0.354	0.787	0.082	4.344	0.450	0.105	0.104
48	0.268	0.642	0.051	5.284	0.417	0.097	0.079
49	0.263	0.660	0.071	3.681	0.399	0.093	0.108
50	0.286	0.688	0.071	4.013	0.416	0.097	0.104
51	0.292	0.737	0.074	3.964	0.396	0.092	0.100
52	0.244	0.644	0.061	4.012	0.379	0.088	0.095
53	0.289	0.725	0.071	4.059	0.398	0.093	0.098
54	0.282	0.704	0.057	4.922	0.401	0.093	0.081
55	0.302	0.711	0.075	4.032	0.425	0.099	0.105
56	0.244	0.607	0.058	4.236	0.402	0.093	0.095
57	0.247	0.670	0.057	4.328	0.369	0.086	0.085
58	0.298	0.692	0.071	4.196	0.431	0.100	0.103
59	0.374	0.730	0.100	3.754	0.512	0.119	0.136
60	0.263	0.683	0.071	3.706	0.385	0.090	0.104
61	0.291	0.650	0.048	6.025	0.448	0.104	0.074
62	0.206	0.511	0.055	3.747	0.402	0.094	0.107
63	0.263	0.654	0.054	4.882	0.401	0.093	0.082
64	0.224	0.605	0.056	3.996	0.370	0.086	0.093
65	0.260	0.720	0.062	4.169	0.361	0.084	0.087
66	0.289	0.718	0.069	4.160	0.402	0.093	0.097
67	0.267	0.568	0.067	4.017	0.471	0.109	0.117
68	0.279	0.669	0.064	4.334	0.417	0.097	0.096
69	0.269	0.701	0.065	4.145	0.383	0.089	0.092
70	0.231	0.465	0.062	3.724	0.496	0.115	0.133
71	0.282	0.646	0.070	4.031	0.436	0.101	0.108
72	0.305	0.693	0.075	4.077	0.440	0.102	0.108
73	0.298	0.638	0.074	4.046	0.468	0.109	0.116
74	0.224	0.468	0.058	3.892	0.480	0.112	0.123
75	0.305	0.669	0.070	4.377	0.456	0.106	0.104
76	0.284	0.708	0.064	4.411	0.401	0.093	0.091
77	0.341	0.787	0.081	4.183	0.433	0.101	0.104
78	0.289	0.633	0.071	4.074	0.457	0.106	0.112
79	0.291	0.662	0.065	4.477	0.440	0.102	0.098
80	0.266	0.676	0.059	4.489	0.393	0.091	0.088
81	0.320	0.706	0.073	4.390	0.452	0.105	0.103
82	0.292	0.633	0.071	4.103	0.461	0.107	0.112
83	0.328	0.768	0.079	4.165	0.427	0.099	0.103
84	0.290	0.734	0.047	6.158	0.395	0.092	0.064
85	0.235	0.514	0.061	3.874	0.457	0.106	0.118
86	0.237	0.511	0.062	3.835	0.463	0.108	0.121
Mean	0.294	0.686	0.072	4.150	0.428	0.100	0.105
*σ*	0.051	0.094	0.016	0.590	0.041	0.009	0.020
CV	0.174	0.136	0.221	0.142	0.095	0.095	0.185

**Table 18:** Abundance of PAAS determined in HTP mode for comparison (CBB stands for the Bradford assay).

No.	PAAS Activity (kU/L)	PAAS quantity by CBB (g/L)	PAAS quantity by ITA (g/L)	PAAS specific activity based on ITA (kU/g)	PAAS apparent specific activity by CBB (kU/g)	PAAS abundance by activity (its known specific activity used)	PAAS abundance by ITA
1	0.847	0.534	0.059	14.307	1.587	0.109	0.111
2	1.264	0.758	0.089	14.253	1.667	0.115	0.117
3	1.130	0.675	0.082	13.858	1.674	0.115	0.121
4	0.853	0.521	0.062	13.693	1.638	0.113	0.120
5	0.685	0.474	0.050	13.755	1.445	0.100	0.105
6	0.804	0.573	0.061	13.151	1.403	0.097	0.107
7	0.805	0.487	0.056	14.357	1.654	0.114	0.115
8	0.818	0.615	0.063	12.916	1.328	0.092	0.103
9	1.031	0.670	0.070	14.727	1.538	0.106	0.104
10	0.511	0.311	0.033	15.253	1.642	0.113	0.108
11	0.554	0.337	0.041	13.447	1.643	0.113	0.122
12	1.111	0.688	0.073	15.262	1.614	0.111	0.106
13	0.717	0.471	0.051	14.166	1.522	0.105	0.107
14	0.900	0.559	0.065	13.863	1.610	0.111	0.116
15	0.743	0.453	0.051	14.663	1.641	0.113	0.112
16	0.724	0.458	0.045	16.185	1.580	0.109	0.098
17	0.796	0.535	0.052	15.212	1.488	0.103	0.098
18	0.865	0.579	0.066	13.110	1.493	0.103	0.114
19	0.712	0.454	0.048	14.945	1.568	0.108	0.105
20	0.769	0.630	0.062	12.402	1.221	0.084	0.098
21	0.712	0.464	0.048	14.758	1.535	0.106	0.104
22	0.960	0.598	0.068	14.080	1.606	0.111	0.114
23	0.984	0.608	0.067	14.759	1.619	0.112	0.110
24	0.770	0.462	0.049	15.648	1.666	0.115	0.106
25	1.087	0.651	0.083	13.073	1.669	0.115	0.128
26	0.843	0.570	0.066	12.738	1.479	0.102	0.116
27	0.766	0.472	0.052	14.784	1.623	0.112	0.110
28	0.962	0.596	0.072	13.363	1.614	0.111	0.121
29	0.959	0.554	0.069	13.856	1.730	0.119	0.125
30	0.922	0.567	0.067	13.813	1.627	0.112	0.118
31	0.920	0.592	0.076	12.078	1.553	0.107	0.129
32	0.835	0.604	0.063	13.304	1.383	0.095	0.104
33	0.748	0.439	0.058	12.972	1.703	0.117	0.131
34	0.812	0.520	0.057	14.150	1.561	0.108	0.110
35	0.829	0.637	0.060	13.908	1.300	0.090	0.093
36	0.821	0.484	0.057	14.315	1.695	0.117	0.118
37	0.858	0.497	0.061	14.109	1.726	0.119	0.122
38	0.848	0.482	0.057	14.798	1.760	0.121	0.119
39	1.062	0.655	0.083	12.792	1.622	0.112	0.127
40	0.748	0.455	0.057	13.074	1.643	0.113	0.126
41	0.820	0.479	0.056	14.730	1.712	0.118	0.116
42	0.760	0.513	0.053	14.325	1.482	0.102	0.103
43	0.793	0.545	0.051	15.552	1.455	0.100	0.094
44	0.763	0.467	0.051	14.854	1.634	0.113	0.110
45	0.800	0.480	0.056	14.257	1.667	0.115	0.117
46	0.867	0.496	0.061	14.268	1.748	0.121	0.123
47	0.848	0.613	0.060	14.191	1.384	0.095	0.098
48	0.866	0.502	0.056	15.490	1.725	0.119	0.111
49	0.806	0.547	0.051	15.832	1.474	0.102	0.093
50	0.968	0.653	0.067	14.481	1.484	0.102	0.102
51	0.821	0.513	0.057	14.371	1.601	0.110	0.111
52	0.827	0.605	0.052	16.034	1.367	0.094	0.085
53	0.909	0.546	0.059	15.423	1.664	0.115	0.108
54	0.830	0.542	0.057	14.469	1.531	0.106	0.106
55	0.932	0.689	0.064	14.475	1.354	0.093	0.094
56	1.095	0.893	0.078	13.964	1.226	0.085	0.088
57	1.412	0.900	0.098	14.364	1.568	0.108	0.109
58	1.210	0.759	0.069	17.646	1.594	0.110	0.090
59	0.763	0.696	0.055	13.985	1.097	0.076	0.078
60	0.888	0.796	0.052	17.055	1.116	0.077	0.065
61	0.879	0.693	0.074	11.858	1.269	0.088	0.107
62	0.945	0.687	0.055	17.244	1.375	0.095	0.080
63	1.917	0.810	0.121	15.908	2.366	0.163	0.149
64	1.985	0.801	0.119	16.644	2.477	0.171	0.149
65	1.636	0.825	0.115	14.175	1.984	0.137	0.140
66	1.331	0.721	0.115	11.547	1.847	0.127	0.160
67	1.030	0.671	0.074	13.913	1.534	0.106	0.110
68	0.941	0.643	0.076	12.387	1.462	0.101	0.118
69	1.323	0.876	0.102	12.964	1.510	0.104	0.116
70	1.377	0.825	0.103	13.352	1.670	0.115	0.125
71	1.966	0.875	0.120	16.371	2.248	0.155	0.137
72	1.608	0.828	0.115	13.938	1.943	0.134	0.139
73	1.116	0.686	0.063	17.853	1.627	0.112	0.091
74	1.184	0.753	0.102	11.562	1.572	0.108	0.136
75	1.031	0.734	0.094	10.914	1.404	0.097	0.129
76	0.961	0.668	0.077	12.518	1.439	0.099	0.115
77	1.448	0.796	0.105	13.793	1.821	0.126	0.132
78	1.561	0.822	0.115	13.611	1.899	0.131	0.140
79	1.564	0.831	0.121	12.900	1.883	0.130	0.146
80	1.201	0.702	0.090	13.276	1.711	0.118	0.129
81	1.192	0.747	0.086	13.825	1.596	0.110	0.115
82	0.779	0.472	0.065	12.009	1.651	0.114	0.137
83	1.005	0.708	0.070	14.330	1.419	0.098	0.099
84	0.794	0.506	0.054	14.665	1.571	0.108	0.107
85	1.096	0.656	0.068	16.011	1.670	0.115	0.104
86	0.864	0.633	0.062	13.979	1.365	0.094	0.098
87	0.878	0.472	0.061	14.473	1.860	0.128	0.129
88	0.847	0.490	0.053	15.960	1.731	0.119	0.108
89	1.235	0.777	0.068	18.293	1.591	0.110	0.087
90	1.151	0.738	0.068	16.865	1.558	0.107	0.092
91	0.808	0.569	0.051	15.947	1.422	0.098	0.089
92	1.297	0.788	0.074	17.452	1.645	0.113	0.094
Mean	0.989	0.617	0.069	14.350	1.599	0.110	0.112
*σ*	0.291	0.133	0.020	1.453	0.218	0.015	0.017
CV	0.294	0.216	0.295	0.101	0.136	0.136	0.151

**Table 19:** Abundance of PAAS/mutants estimated by two ways with 48-well microplate.

PAAS/mutants (48-well)	PAAS	G138S	M72D	M72Q
Specific activity after purification by microplate reader	14.6	3.4	2.3	4.3
Ratio of specific activity to M72D	6.35	1.48	1.00	1.87
*n* for independent lysates	92	80	90	86
Activity concentration in lysates (kU/L)	0.94±0.29	0.25±0.07	0.21±0.05	0.29±0.05
Ratio of activity concentration to M72D	4.48	1.19	1.00	1.38
Total protein (g/L)	0.6±0.1	0.7±0.2	0.8±0.1	0.7±0.1
Apparent specific activity based on total proteins (kU/g)	1.6±0.2	0.4±0.1	0.3±0.0	0.4±0.0
Ratio of apparent specific activity to M72D	6.1	1.4	1.0	1.7
Protein by ITA (g/L)	0.06±0.02	0.07±0.03	0.09±0.03	0.07±0.02
Specific activity based on ITA (kU/g)	14.60±1.5	3.67±0.69	2.39±0.31	4.15±0.59
Ratio of specific activity to M72D	6.10	1.54	1.00	1.74
Abundance by known specific activity	(11±1)%	(11±2)%	(11±2)%	(10±1)%
Abundance by ITA	(11±2)%	(10±3)%	(11±3)%	(11±2)%

Initial rate of each enzyme was estimated with data from 10 to 15 min reaction with BIOTEK ELX 800 microplate reader. Cell lysates were prepared in 0.70 mL lysis buffer with cell suspension of 0.40 mL (0.50 mL medium was used with 48-well microplate).

**Table 20:** Abundance of PAAS/mutants estimated by two ways with cells amplified in 4.0 mL medium to prepare 2.5 mL lysate.

PAAS/mutants	PAAS	G138S	M72D	M72Q
Specific activity after purification by microplate reader	14.6	3.4	2.3	4.3
Ratio of specific activity to M72D	6.35	1.48	1.00	1.87
*n* for independent lysates	13	12	10	10
Activity concentration (kU/L)	3.64±2.34	0.69±0.43	0.48±0.28	0.89±0.53
Ratio of activity concentration to M72D	7.58	1.44	1.00	1.84
Total protein ( g/L)	1.3±0.5	1.2±0.5	1.2±0.6	1.2±0.6
Apparent specific activity based on total protein (kU/g)	2.8±1.8	0.6±0.4	0.4±0.3	0.8±0.6
Ratio of apparent specific activity to M72D	7.2	1.5	1.0	2.0
Protein quantity by ITA (g/L)	0.25±0.07	0.19±0.06	0.22±0.06	0.22±0.07
Specific activity based on ITA (kU/g)	13.48±2.5	3.63±0.69	2.18±0.41	4.05±0.89
Ratio of specific activity based on ITA to M72D	6.18	1.67	1.00	1.86
Abundance by known specific activity	(19±5)%	(17±4)%	(17±5)%	(18±5)%
Abundance by ITA	(19±3)%	(16±4)%	(18±4)%	(19±3)%

Initial rate of each enzyme was estimated with data from 10 to 15 min reaction with BIOTEK ELX 800 microplate reader. Cell lysates were prepared in 2.5 mL lysis buffer with cell suspension of 4.0 mL medium.

**Table 21:** Abundance of BFU/mutants estimated by two ways with cells amplified in 4.0 mL medium to prepare 2.5 mL lysate.

BFU/mutants (4.0 mL)	BFU	BFU-6H	A1R	F301L-6H	L171I
Specific activity by microplate after purification and the correction of purity	9.0	3.5	3.3	1.6	2.6
Ratio of specific activity to F301L-6H	5.59	2.17	2.05	1.00	1.61
*n* for independent lysates	10	8	5	3	4
Activity concentration in cell lysates (kU/L)	1.69±0.59	0.83±0.37	0.70±0.42	0.33±0.31	0.56±0.32
Ratio of activity concentration to F301L-6H	5.12	2.52	2.12	1.00	1.52
Total proteins (g/L)	1.3±0.2	1.4±0.3	1.4±0.4	1.3±0.31	1.3±0.4
Specific activity based on total proteins (kU/g)	1.3±0.5	0.6±0.3	0.5±0.3	0.3±0.2	0.4±0.3
Ratio of activity concentration to F301L-6H	5.2	2.4	2.1	1.0	1.7
Protein quantity by ITA (g/L)	0.23±0.22	0.24±0.23	0.22±0.32	0.24±0.44	0.23±0.35
Specific activity based on ITA (kU/g)	6.26±1.32	3.07±0.96	2.69±1.38	1.18±0.85	2.07±1.5
Ratio of specific activity based on ITA to F301L-6H	5.31	2.60	2.28	1.00	1.76
Abundance by known specific activity	(16±6)%	(17±6)%	(16±6)%	(17±7)%	(17±6)%
Abundance by ITA	(17±3)%	(18±3)%	(16±4)%	(17±3)%	(18±3)%

Initial rate was estimated with data from 10 to 15 min reaction with BIOTEK EON microplate reader.

**Table 22:** Abundance of PAAS/mutants estimated by two ways with cells amplified in 250 mL medium to prepare 50 mL lysate.

PAAS/mutants	PAAS	G138S	M72D	M72Q
Specific activity after purification by microplate reader	14.6	3.4	2.3	4.3
Ratio of specific activity to M72D	6.35	1.48	1.00	1.87
*n* for independent lysates	9	8	8	8
Activity concentration (kU/L)	12.37±2.67	4.73±1.06	2.09±0.48	4.45±1.04
Ratio of activity concentration to M72D	12.28	2.25	1.00	4.27
Total protein ( g/L)	4.7±1.1	5.4±1.3	4.2±0.9	4.7±1.1
Apparent specific activity based on total protein (kU/g)	2.7±0.6	0.9±0.2	0.5±0.1	1.0±0.2
Ratio of apparent specific activity to M72D	5.6	1.8	1.0	1.9
Protein quantity by ITA (g/L)	0.96±0.20	0.93±0.19	0.86±0.17	0.87±0.17
Specific activity based on ITA (kU/g)	12.89±2.91	5.10±0.48	2.43±0.50	5.10±0.33
Ratio of specific activity based on ITA to M72D	5.30	2.10	1.00	2.10
Abundance by known specific activity	(21±5)%	(15±4)%	(22±5)%	(18±5)%
Abundance by ITA	(21±4)%	(21±4)%	(20±4)%	(19±3)%

Initial rate of each enzyme was estimated with data from 10 to 15 min reaction with BIOTEK ELX 800 microplate reader. Cell lysates were prepared in 50 mL lysis buffer with cell suspension of 250 mL medium.

**Table 23:** Abundance of BFU/mutants estimated by two ways with cells amplified in 250 mL medium to prepare 50 mL lysate.

BFU/mutants (4.0 mL)	BFU	BFU-6H	A1R	F301L-6H	L171I
Specific activity by microplate after purification and the correction of purity	9.0	3.5	3.3	1.61	2.60
Ratio of specific activity to F301L-6H	5.59	2.17	2.05	1.00	1.61
*n* for independent lysates	7	6	5	4	3
Activity concentration in cell lysates (kU/L)	5.52±1.39	2.14±0.54	2.05±0.52	0.99±0.25	1.56±0.39
Ratio of activity concentration to F301L-6H	5.58	2.16	2.07	1.00	1.58
Total proteins (g/L)	5.3±1.1	5.2±1.1	5.2±1.1	5.2±1.1	5.3±1.1
Specific activity based on total proteins (kU/g)	1.1±0.2	0.4±0.1	0.4±0.1	0.2±0.0	0.3±0.1
Ratio of activity concentration to F301L-6H	5.5	2.2	2.1	1.0	1.6
Protein quantity by ITA (g/L)	0.92±0.22	0.94±0.23	0.92±0.32	0.95±0.44	0.98±0.35
Specific activity based on ITA (kU/g)	6.00±1.28	2.28±0.56	2.23±0.48	1.04±0.23	1.59±0.38
Ratio of specific activity based on ITA to F301L-6H	5.77	2.19	2.14	1.00	1.53
Abundance by known specific activity	(18±4)%	(18±4)%	(18±5)%	(18±4)%	(19±5)%
Abundance by ITA	(18±4)%	(18±4)%	(18±4)%	(18±3)%	(19±4)%

Initial rate was estimated with data from 10 to 15 min reaction with BIOTEK EON microplate reader.

**Table 24:** Examination of consistence of accessible epitopes on enzyme/mutants.

Enzyme/mutants^a^		Specific activity after purification^b^	Abundance derived from activity (%)	Abundance by ITA (%)
PAAS/mutants (48-well)	PAAS (92)	14.6	11±1	11±2
	M72Q (80)	4.3	10±1	11±2
	G138S (90)	3.4	11±2	10±3
	M72D (89)	2.3	11±2	11±3
PAAS/mutants^c^ (4.0 mL)	PAAS (13)	14.6	19±5	19±3
	M72Q (12)	4.3	17±4	16±3
	G138S (10)	3.4	17±4	18±4
	M72D (10)	2.3	18±5	19±4
BFU/ mutants^c^ (4.0 mL)	BFU (10)	9.0	16±6	17±3
	BFU-6H (8)	3.5	17±6	18±3
	A1R (5)	3.3	16±6	16±4
	L171I (3)	2.6	17±7	17±3
	F301L-6H (4)	1.6	17±6	18±3
PAAS/mutants^c^ (250 mL)	PAAS (13)	14.6	21±5	21±4
	M72Q (12)	4.3	18±4	19±3
	G138S (10)	3.4	15±4	21±4
	M72D (10)	2.3	22±5	20±4
BFU/ mutants^c^ (250 mL)	BFU (10)	9.0	18±4	18±4
	BFU-6H (8)	3.5	18±4	18±4
	A1R (5)	3.3	18±5	18±4
	L171I (3)	2.6	18±4	19±4
	F301L-6H (4)	1.6	19±5	18±3

a Number in parenthesis indicated independent lysates, and all samples suitable for ITA were analyzed. For each enzyme/mutant, paired *t*-test comparison supported no difference for the abundance derived from activity and by ITA. There were no differences among abundance derived from activity or by ITA in each group of enzyme/mutants under stated conditions.

b Each specific activity after purification was expressed in kU/g, with CV<15% from three independent preparations of each enzyme.

c All the cell lysates prepared from 4.0 or 250 mL medium for the amplification of cells and induced expression were included for analyses.

**Table 25:** Associations of specific activities based on ITA with those determined after purification.

PAAS/mutants (4.0 mL)	PAAS	G138S	M72D	M72Q	
Specific activity after purification and the correction of purity and Ni^2+^ inhibition by microplate reader	14.6	3.4	2.3	4.3	
Ratio of specific activity to M72D	6.4	1.5	1.0	1.9	
Specific activity in cell lysate based on ITA (kU/g)	12.9±2.9	5.1±0.5	2.4±0.5	5.1±0.3	
Ratio of specific activity based on ITA to M72D	5.3	2.1	1.0	2.1	
BFU/mutants (4.0 mL)	BFU	BFU-6H	A1R	F301L-6H	L171I
Specific activity after purification and the correction of purity by microplate reader	9.0	3.5	3.3	1.6	2.6
Ratio of specific activity to F301L-6H	5.6	2.2	2.1	1.0	1.6
Specific activity in cell lysates based on ITA (kU/g)	6.0±1.3	2.3±0.6	2.2±0.5	1.0±0.2	1.6±0.4
ratio of specific activity based on ITA to F301L-6H	5.8	2.2	2.1	1.0	1.5

**Table 26:** Summary of AUC by ROC analysis.

Enzyme pairs	Ratio of specific activity	AUC		
		Activity concentration	Apparent specific activity	Specific activity based on ITA
M72Q vs G138S	1.26	0.719	0.782	0.797
G138S vs M72D	1.48	0.726	0.790	0.922
M72Q vs M72D	1.87	0.797	0.864	0.998
PAAS vs M72Q	3.37	0.997	1.000	1.000
PAAS vs G138S	4.26	1.000	1.000	1.000
PAAS vs M72D	6.30	1.000	1.000	1.000

## 2 Experimental design, materials and methods

### 2.1 Experimental design

The proposed strategy requires consistent accessible epitopes among enzyme/mutants for the use of just one reference [1].

The actions of PAAS/mutants and BFU/mutants do not alter cell growth and they should have consistent abundance among intracellular proteins after induced expression under the same conditions. They served as two models to test the validity of the proposed strategy.

The abundance of an enzyme/mutant based on ITA was the ratio of its protein quantity determined by ITA to that of total proteins; the abundance of an enzyme/mutant by activity was the ratio of its apparent specific activity to its specific activity after careful purification.

For a group of PAAS/mutants or BFU/mutants, they should have consistent accessible epitopes when their abundance based on ITA with just one reference is consistent with each other and further consistent with those by activities.

### 2.2 Materials and methods

All experiments involving rabbits were approved by the Animal Care and Use Committee of Chongqing Medical University (China).

For each enzyme/mutant, a individual clone was amplified at 37 °C and 180 rpm, for 12 h with 0.50 mL of the medium in 48-well microplates, but for 4.0 h with 4.0 mL or 250 mL of the medium in glass bottles. Each enzyme/mutant was induced by 1.0 mM isopropyl-β-*D*-thiogalactoside at 16 °C and 110 rpm for 20 h. Cells were broken by sonication treatment in 20 mM Tris–HCl at pH 8.0; 0.50 mL of the cell lysate after centrifugation at 10,000 rpm for 15 min was filtered through 0.22-μm membrane to serve as a sample for both ITA and activity assay [1], [2].

With PAAS/mutants, 2.0 mM potassium 4-nitrophenylsulfate was used in 1.0 mM Tris–HCl at pH 9.0 to measure the absorbance at 405 nm with Biotek ELX 800 [1], [3]. With BFU/mutants, 0.14 mM uric acid was employed in 0.20 M sodium borate at pH 9.2 to measure absorbance at 293 nm with Biotek EON [1], [4]. Their initial rates were derived from absorbance change from 10.0 to 15.0 min since reaction initiation [1]. The apparent specific activity was the ratio of the activity to the quantity of total proteins in an unpurified sample.

ITA quantified the difference of the extinction at 370 and 700 nm for reaction mixture of 0.20 mL in 96-well microplates with Biotek EON.

Ratios of their specific activities were calculated with the specific activities of 14.6, 4.3, 3.4, 2.3 kU/g for PAAS, M72Q, G138S and M72D, respectively.

## Figures and Tables

**Fig. S1:**
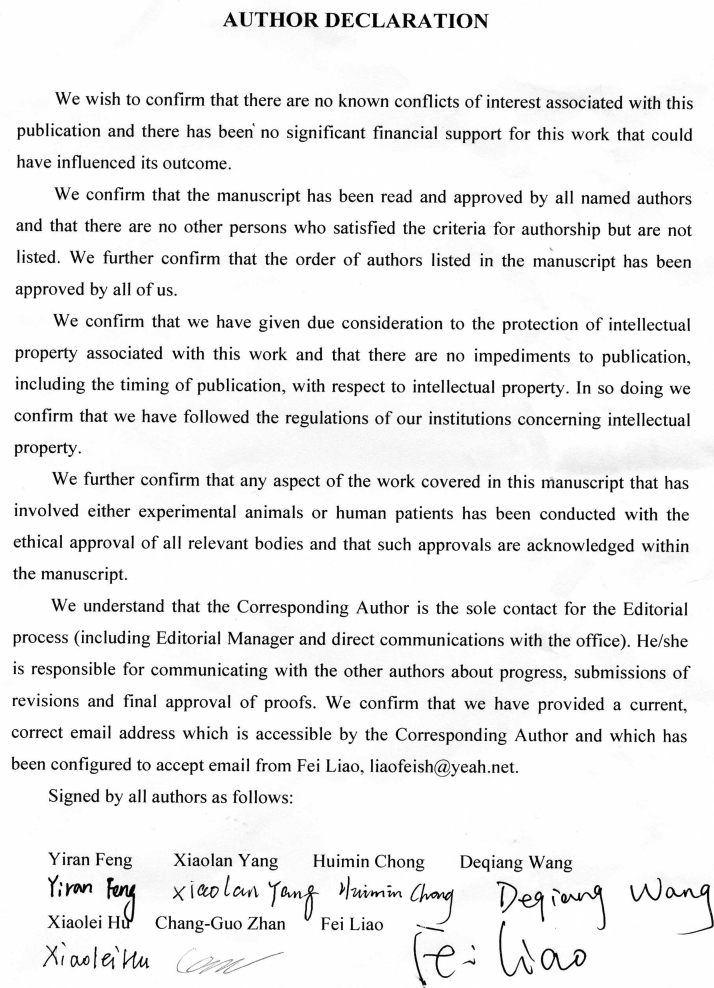
.
